# Effect of different physical factors on the synthesis of spherical gold nanoparticles towards cost‐effective biomedical applications

**DOI:** 10.1049/nbt2.12100

**Published:** 2022-11-03

**Authors:** Zahra Bahmanyar, Fatemeh Mohammadi, Ahmad Gholami, Mehdi Khoshneviszadeh

**Affiliations:** ^1^ Department of Pharmaceutical Biotechnology School of Pharmacy Shiraz University of Medical Sciences Shiraz Iran; ^2^ Biotechnology Research Center Shiraz University of Medical Sciences Shiraz Iran; ^3^ Pharmaceutical Sciences Research Center Shiraz University of Medical Sciences Shiraz Iran; ^4^ Department of Medicinal Chemistry School of Pharmacy Shiraz University of Medical Sciences Shiraz Iran; ^5^ Medicinal and Natural Products Chemistry Research Center Shiraz University of Medical Sciences Shiraz Iran

**Keywords:** gold nanoparticles, synthesis efficacy, synthesis optimization, synthesis parameters, Turkevich method

## Abstract

Gold nanoparticles (AuNPs) have great potential to contribute to numerous application fields of biomedicine, which are highly dependent on their physicochemical properties, such as size and shape. Due to the final characteristics, nanoparticles (NPs) are primarily affected by different factors of reaction conditions; the present study aimed to evaluate the effects of manipulating the main physical parameters of the Turkevich method to optimise the fabrication of citrated capped AuNPs in a spherical shape, desirable final size, and efficiency. For this purpose, various experiments of citrate‐capped spherical AuNPs synthesis were designed to study the roles of a wide range of initial pH values and temperature of reaction, Na_3_Cit/HAuCl_4_ molar ratio, and two order reagent additions, method I and method II, in the final characterisations and reaction efficacy. Prepared NPs synthesised with different experiments were characterised by dynamic light scattering, UV‐Visible, and fourier transform infrared spectroscopy. Furthermore, NPs obtained from optimised synthesis conditions were more detailed using UV‐Visible, transmission electron microscopy, and XRD. The findings indicated that the final size and synthesis efficacy of citrated capped spherical AuNPs were significantly affected by all studied synthesis parameters and the order addition of reagents. The higher initial reaction temperature and Na_3_Cit/HAuCl_4_ Molar ratio provided a smaller particle size with desirable synthesis efficacy. Besides, final optimised NPs were provided in cubic crystal structures, and each NP's single crystal was obtained. In sum, our findings indicated that optimising synthesis conditions could improve size distribution, morphology, crystallite size, and structures of final NPS, as well as efficiency, which is a principal factor associated with future cost‐effective productions on large scales. Further studies are needed in this regard.

## Graphical abstract

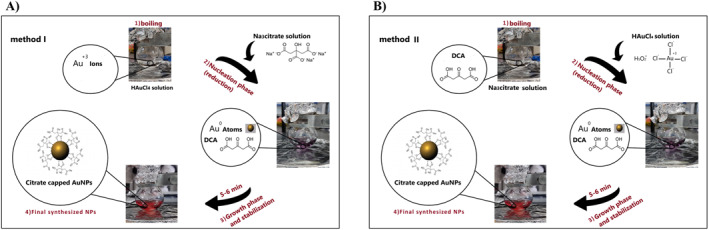

In this study, citrated‐capped spherical AuNPs were fabricated by numerous designed synthesis experiments based on the Terkevich method, in order to optimization of synthesis conditions to achieve a final desirable particle size along with acceptable synthesis efficacy and productivity toward biological applications. Due to the final characteristics of these NPs are largely affected by different factors of reaction conditions, the main physical parameters of Terkevich approach were manipulated to evaluate their impacts in details including a wide range of initial temperature, initial pH of reaction, and a various range of Trisodium citrate/HAuCl4 molar ratios. Moreover, given the significant impact of the reagent addition order on final particles' characteristics and synthesis efficiency, all experiments were also performed in two different addition orders of reagents, HAuCl4 and Trisodium citrate salt to study more detailed factors.

## 1 INTRODUCTION

Recently, gold nanoparticles (AuNPs) have attracted considerable biomedical interest in high biocompatibility, physicochemical properties, and characteristics tunability in synthesis [1, 2]. Gold nanoparticles have been studied in a wide range of biomedicine applications from diagnosis to treatment, including biosensors [2], gene and drug delivery [3, 4], phototherapy and hyperthermia [5], and antimicrobial applications [6] in different shapes of nanostars [7], nanorods [5], nanocages [8], and nanosphere [9]. It is widely accepted that physicochemical properties of nanoparticles (NPs), predominantly size and morphology, determine their action inherently in in vitro and in vivo applications [10]; previous findings indicated that the smaller sizes of AuNPs, between 10 and 30 nm inserted easier into cancerous tumour cells than larger sizes [11]. Surface‐coated AuNPs showed more cell uptake in smaller sizes, 20 nm, than 40 and 80 nm [12].

Furthermore, The NPs' size and shape play a fundamental role in long circulation, biodistribution, and releasing drugs in delivery systems, so the smaller size and spherical morphology is a good candidate in this regard. Smaller size NPs allow faster drug release due to the larger surface‐to‐volume ratio and the more potential for cellular uptake of spherical morphology [13]. As reported by previous studies, the shape of NPs is one of the significant determinative factors of desirable applications in biomedicine; since the spherical shapes of AuNPs revealed higher sensitivity and specificity in biosensors [14], while elongation and increase of sharpness of nanostructure made them more favoured in photothermal therapy and imaging due to more substantial near‐infrared absorbance [15].

Although the smaller sizes of AuNPs are typically preferred in various applications, this may present some potential drawbacks. The minimal size of these NPs, under 5 nm, is reported to have higher toxicity due to their chemical reactivity [16]. Moreover, previous findings reported that spherical AuNPs with a size of 1.4 nm could induce oxidative stress, mitochondrial damage, and necrosis in studied cell lines. In contrast, there was no evidence of cell damage for 15 nm spherical AuNPs with the same surface group [17]. Therefore, given that the toxicity of AuNPs is size‐dependent, the AuNPs should be prepared in the optimal and appropriate size for each application type, along with fewer adverse effects. Accordingly, we aimed to design various synthesis experiments to optimise the fabrication of AuNPs with a desirable size of nanospheres and acceptable synthesis productivity towards biological applications. For this purpose, different experiments were designed using the Turkevich method by manipulating reaction conditions of this common synthesis approach, including initial temperature, initial PH of reaction, and a various range of Trisodium citrate/HAuCl_4_ molar ratios. Turkevich method is a relatively convenient and reproducible technique to achieve the small size of gold nanospheres by using Trisodium citrate salt (Na_3_Cit) as a reducing and stabilising agent. In this synthesis method, the reduction of gold chloride salt in aqueous solution results in the synthesis of monodisperse AuNPs suspensions with tunable particle size. In this reaction, to achieve a particle size of less than 20 nm, 1 ml of 1% Na_3_Cit solution should be suddenly added to boiling HAuCl_4_ solution with a concentration of 0.01 by weight. After 5 min, the complete colour change indicates the formation of AuNPs [18]. Moreover, given the significant impact of the additional orders of precursors on final particles' characteristics and synthesis efficiency [19], in the current study, all designed synthesis experiments were also carried out in two different addition orders of reagents, HAuCl_4_ and Na_3_Cit salt.

## 2 MATERIALS AND METHODS

### 2.1 Materials

Tetrachloroauric (III) acid trihydrate (HAuCl_4_.3H_2_O) and Trisodium citrate (Na_3_C_6_H_5_O_7_.2H_2_O) were respectively purchased from Shirazchem Co. and Kimia mavad Co, Iran. Sodium hydroxide (NaOH) and Hydrochloric acid (HCl) were purchased from Sigma Chemical Co., St. Louis, Mo.

### 2.2 Synthesis experiments of AuNPs

Before each synthesis process, the round‐bottom flask was washed with freshly prepared aqua regia acid solution, a mixture of NaOH and HCl with a molar ratio of 1:3, to prevent contamination. Then, 10 ml of deionised water was added to 58 μL of 0.05 M HAuCl_4_. Formerly, the specific concentrations of Na_3_Cit solution were suddenly added to the mixture during vigorously stirring. Constant air pressure is needed before the addition of the reducing agent. The molar ratio of 0.7, 1.4, 2.1, 2.8, 3.6, 4.3, 5, 5.7, 6.5, and 7.2 were considered for Na_3_Cit/HAuCl_4_ in designed experiments. After about 5 min vigorously stirring at boiling temperature and complete colour change, the synthesis process was performed. Due to the size of the final synthesised NPs, this colour change can be in the range of orange‐red to violet. It was gradually cooled to room temperature and finally stored at 4°C. The Na_3_Cit/HAuCl_4_ molar ratio: 0.7–7.2 and initial temperature: 25, 55, 65, 75, 85, 95 and initial pH value: 1–9. The synthesis experiments were performed in two methods: adding the specific concentrations of Na_3_Cit solution to boiling gold salt solution in the method I and adding HAuCl_4_ solution to boiling Na_3_Cit solution in different concentrations, method II.

Since there is a necessity to develop cost‐benefit techniques of NPs synthesis and acceptable efficiency, in this study, the efficiency of different designed experiments was compared with the benefit of the Beer‐Lambert law. Given the Beer‐Lambert law, the final concentration of synthesised AuNPs directly correlated with surface plasmon resonance absorbance in the maximum wavelength [20]; thus, in this study, high absorbance was considered more effective for synthesis (Equation (1)).

(1) A=bCϵ

**A** = absorbance **b** = length of light path **C** = concentration **ϵ** = molar absorptivity.

### 2.3 Characterisation

Gold nanoparticles synthesised with different experiments were characterised by Fourier transform infrared spectroscopy (FTIR Spectroscopy, Vertex 70, Bruker, Germany) to assess their chemical properties. Dynamic light scattering (DLS analyser, Microtrac) to calculate particle size distribution and UV/Visible spectroscopy in the 400–700 nm region (UV/Visible spectrometer, PG Instruments Ltd) to estimate surface plasmon resonance. In addition, the more detailed characterisation tests, including transmission electron microscopy (TEM, Zeiss‐EM10C‐100 kV, Zeiss co) and X‐ray powder diffraction (XRD) analysis (X’ Pert Pro, Panalytical co) were performed for the final AuNPs obtained under optimum synthesis conditions of both methods.

## 3 RESULTS AND DISCUSSION

**FIGURE 1:**
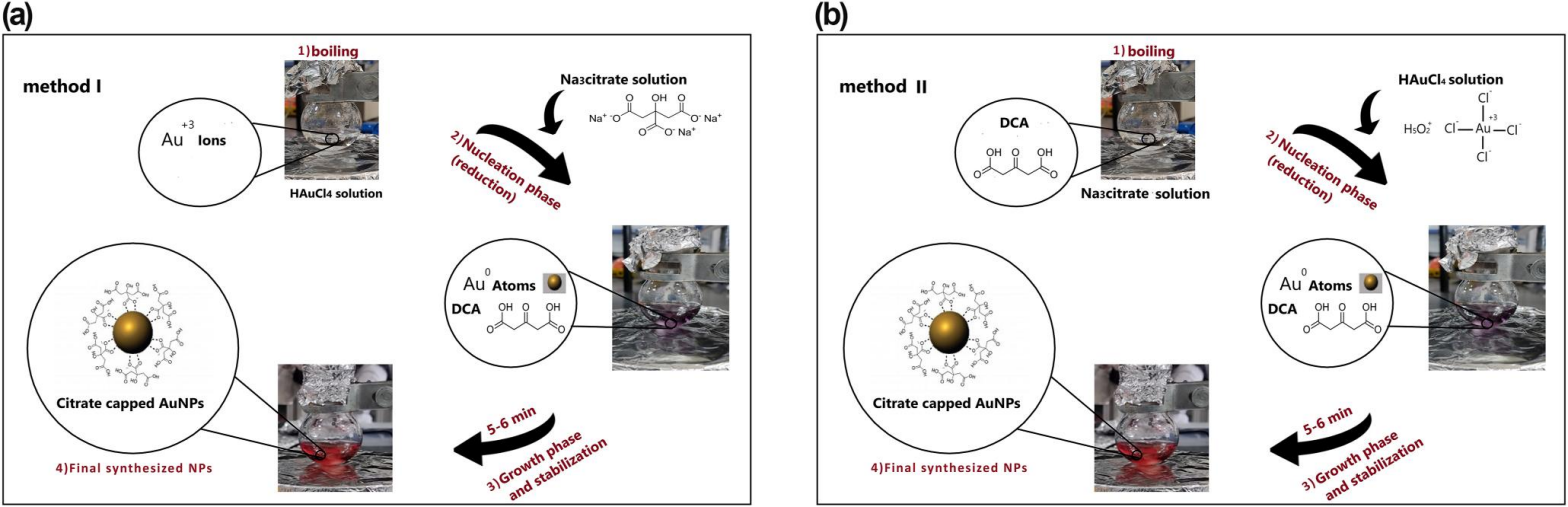
Schematic view for the fabrication of spherical citrate‐capped gold nanoparticles (AuNPs), based on different reagent addition orders of (a) Turkevich approach as method I and (b) Reverse of Turkevich approach as method II.

Turkevich method is one of the practical approaches for the chemical synthesis of spherical citrate‐capped AuNPs due to its convenient procedure and predictable size distribution [18, 21]. In this typical AuNPs synthesis, Na_3_Cit is suddenly added to boiling HAuCl_4_ solution as a reducing and stabilising agent [18]. Figure 1 indicates the synthesis of citrate‐capped AuNPs in detail, based on Turkevich addition order in method I and reverse Turkevich as method II. In both methods, Na_3_Cit initiates nucleation by reducing gold ions; nuclei enter another phase called growth which their sizes increase and turn into seeds that are capped and stabilised by Na_3_Cit. After 5 min of stirring at boiling temperature, the colour change appears, indicating the end of synthesis [18, 22]. In this chemical reduction method, dicarboxylic acid (DCA), as an oxidation product of Na_3_Cit, mainly influences uniformity and controls the size of AuNPs. Dicarboxylic acid is responsible for converting the Au^+3^ ions to Au^0^ atoms, initiating the nucleation phase, and affecting the growth phase and the formation of monodisperse AuNPs [19]. In this survey, the optimisation of AuNPs synthesis was carried out to achieve desired size and efficiency through different designed experiments. The effect of three synthesis parameters, including initial pH and reaction temperature and Na_3_Cit concentration with different molar ratios of Na_3_Cit/HAuCl_4_ 0.7–7.2 in final synthesised NPs, were evaluated. To the importance of the addition order of precursors and its effect on the synthesis process and physicochemical properties of the synthesised NPs, all synthesis experiments were performed in two order addition of reagents, based on Turkevich addition order in the method I and reverse Turkevich as method II.

### 3.1 Characterisations

**FIGURE 2:**
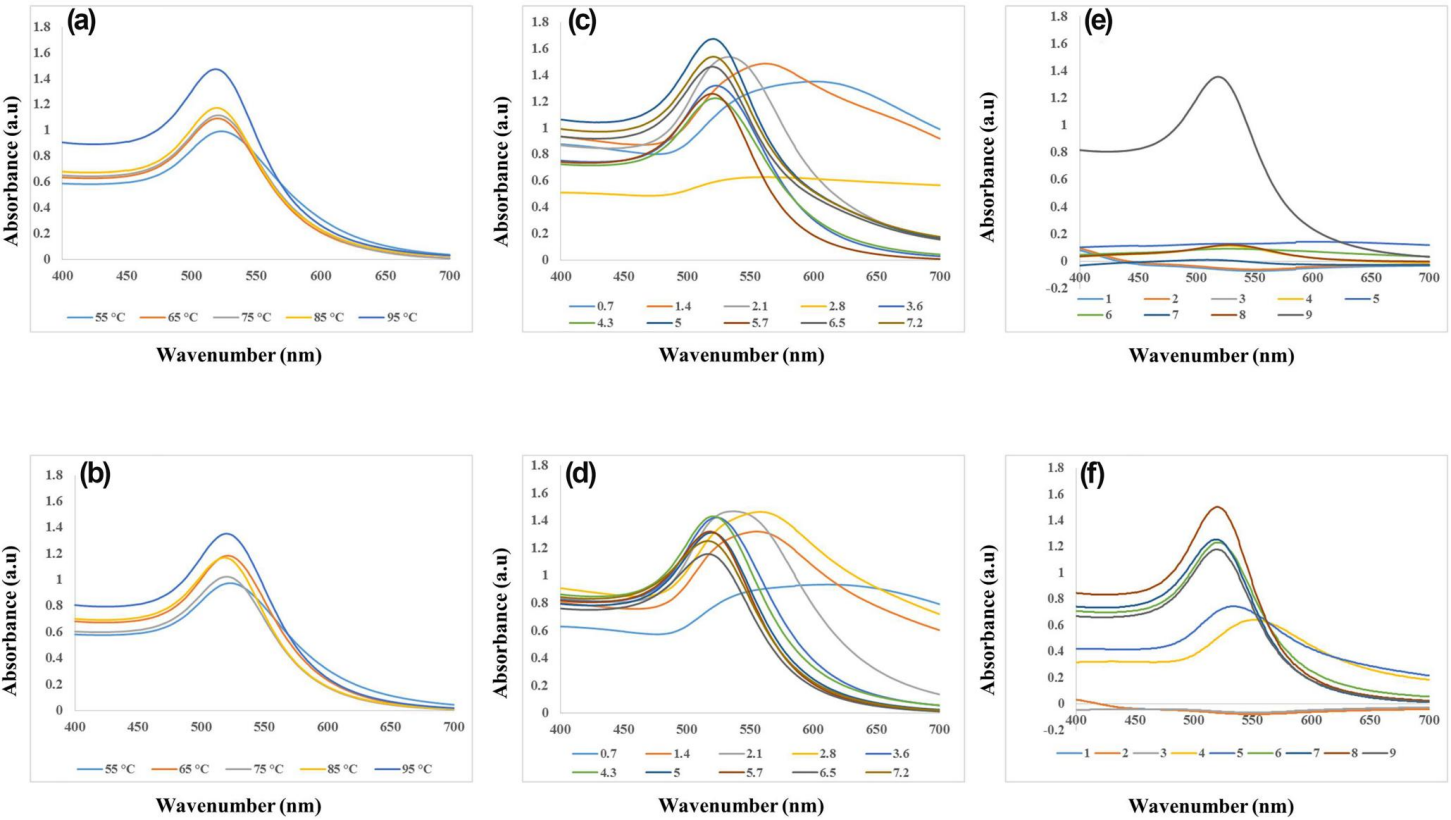
UV‐Vis absorption spectra of citrate‐capped gold nanoparticles (AuNPs) synthesised in different range of (a) and (b) initial temperature, (c) and (d) Na_3_Cit/HAuCl_4_ molar ratio, and (e) and (f) pH values by method I and method II respectively.

Figure 2 shows the UV absorption spectra of citrated capped AuNPs synthesised in numerous experiments designed in a different range of initial temperature, PH, and Na_3_Cit/HAuCl_4_ molar ratio by two methods, I and II. The UV absorption spectrums of all prepared NPs primarily reveal a single absorption peak at 510–570 nm in the visible region ascribed to the characteristic surface plasmon resonance (SPR) absorption of spherical AuNPs [23]. As indicated by previous studies, gold nanospheres typically exhibited just a single SPR band at 520–540 nm in the particle size range of 2–50 nm. In contrast, two SPR bands appeared in UV absorption spectrums of non‐spherical AuNPs [24, 25]. Similar results were also reported for the synthesis of spherical AuNPs in the presence of sodium citrate, which found maximum SPR peaks at 517 nm for the final particle size of 5–10 nm [26].

**FIGURE 3:**
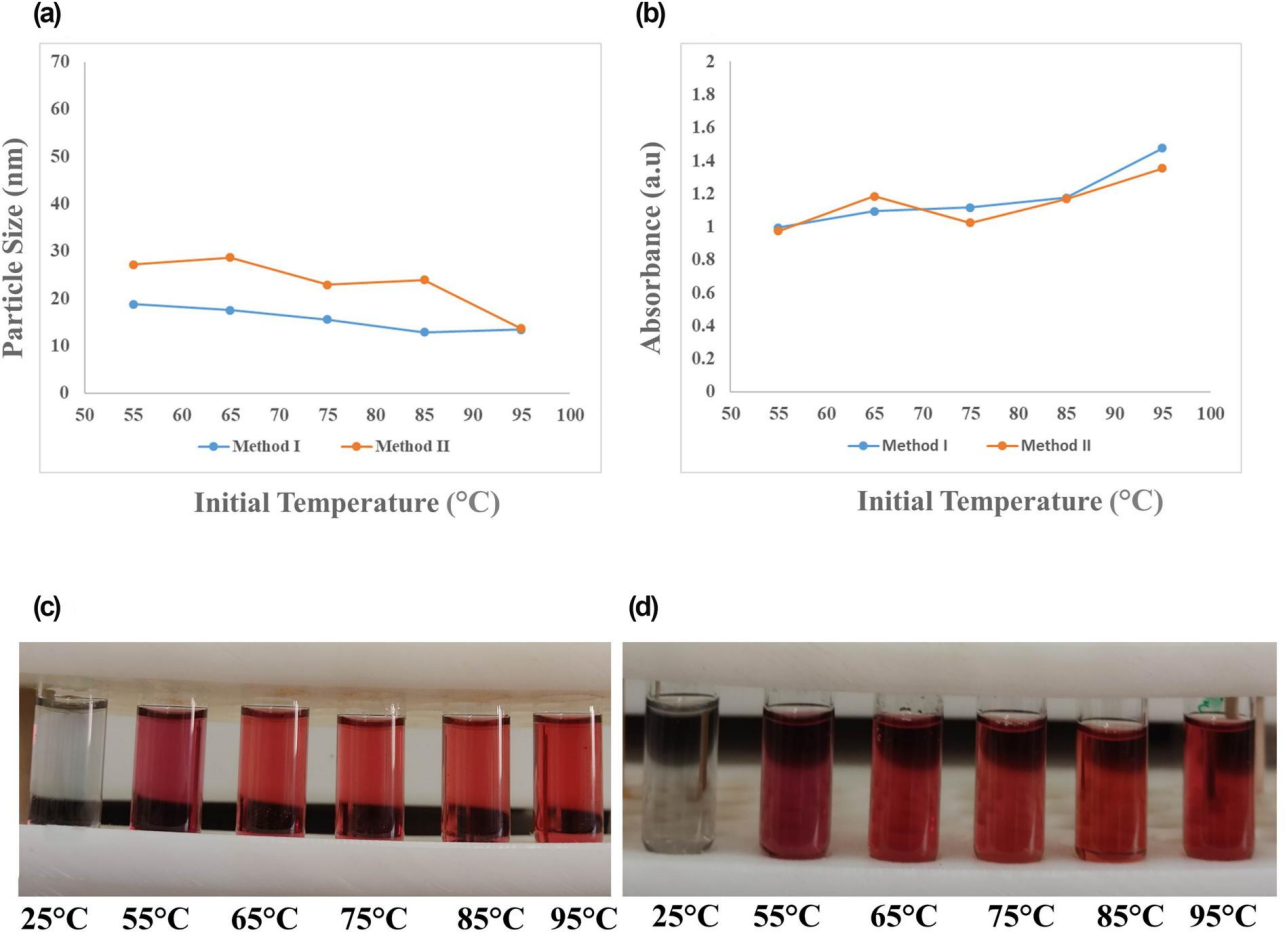
Effect of initial reaction temperature of 25, 55, 65, 75, 85, and 95°C on (a) final particle size and (b) maximum SPR absorption of gold nanoparticles (AuNPs) synthesised by two methods, along with the laboratory images of NPs obtained by (c) method I and (d) method II. SPR, surface plasmon resonance

SPR spectrum of AuNPs obtained from synthesis experiments in the tested range of initial temperature except for 25°C; exhibits the curves with a narrow and sharp peak around 520 nm in both methods (Figure 2a,b). The SPR curves of NPs obtained from the experiment with a temperature of 25°C did not show any peaks, which confirmed the necessity of heating to synthesise AuNPs as the colourless solution is observed in laboratory images at room temperature (Figure 3). Moreover, the results indicated that with rising initial temperature, the peak intensity increases in methods I and II.

Figure 2c,d reveals the SPR spectrum of AuNPs synthesised in different Na_3_Cit/HAuCl_4_ molar ratios in methods I and II. The SPR spectrums of all synthesised AuNPs showed a single peak at 500–600 nm. The experiments with a Na_3_Cit/HAuCl_4_ ratio of >3.6 presented single narrow SPR peaks of final NPs at about 520 and high intensity in both methods. The SPR intensity of produced AuNPs inside the final colloidal solution increased along with decreasing particle size due to rising Na_3_Cit concentrations, which is in line with previous studies [27]. On the other hand, we observed the peak became wider and shifted towards the blue region as decreasing ratio and intensity decreased in experiments with Na_3_Cit/HAuCl_4_ molar ratios of ≤3.6 associated with the rise in particle size of gold nanospheres [28]. It was more than 20 nm in the Na_3_Cit/HAuCl_4_ molar ratio of ≤3.6, as detailed in our findings which confirmed the significant role of Na_3_Cit concentrations in AuNPs fabrication and size distribution [29].

Figure 2e and f demonstrate the effect of different initial pH values of reaction on UV absorption of final AuNPs in both methods. The SPR curves of pH <3 do not present any peaks in both methods that confirmed failure to fabricate AuNPs in high initial acidic pH. A sharp and narrow peak is highlighted in the SPR curve of the experiment with initial pH = 3 in method I, with a good intensity and maximum wavelength absorbance of 519 nm. While in other initial pH values in which synthesis has occurred, including pH values of 4, 8, and 9, the peak intensity remarkably decreased and shifted towards the blue region, *λ*_max_ = 520–550 nm (Figure 2e). As shown in Figure 2f, the SPR spectrum of experiments with initial pH values of 6–9 present sharp and narrow peaks at about 520 nm with fairly good intensity in method II. However, as we increase the pH value to 8 and 9, the intensity of SPR peaks related to produced AuNPs decreases, and the blue shift of the absorption is presented (*λ*_max_ = 530–550 nm).

**FIGURE 4:**
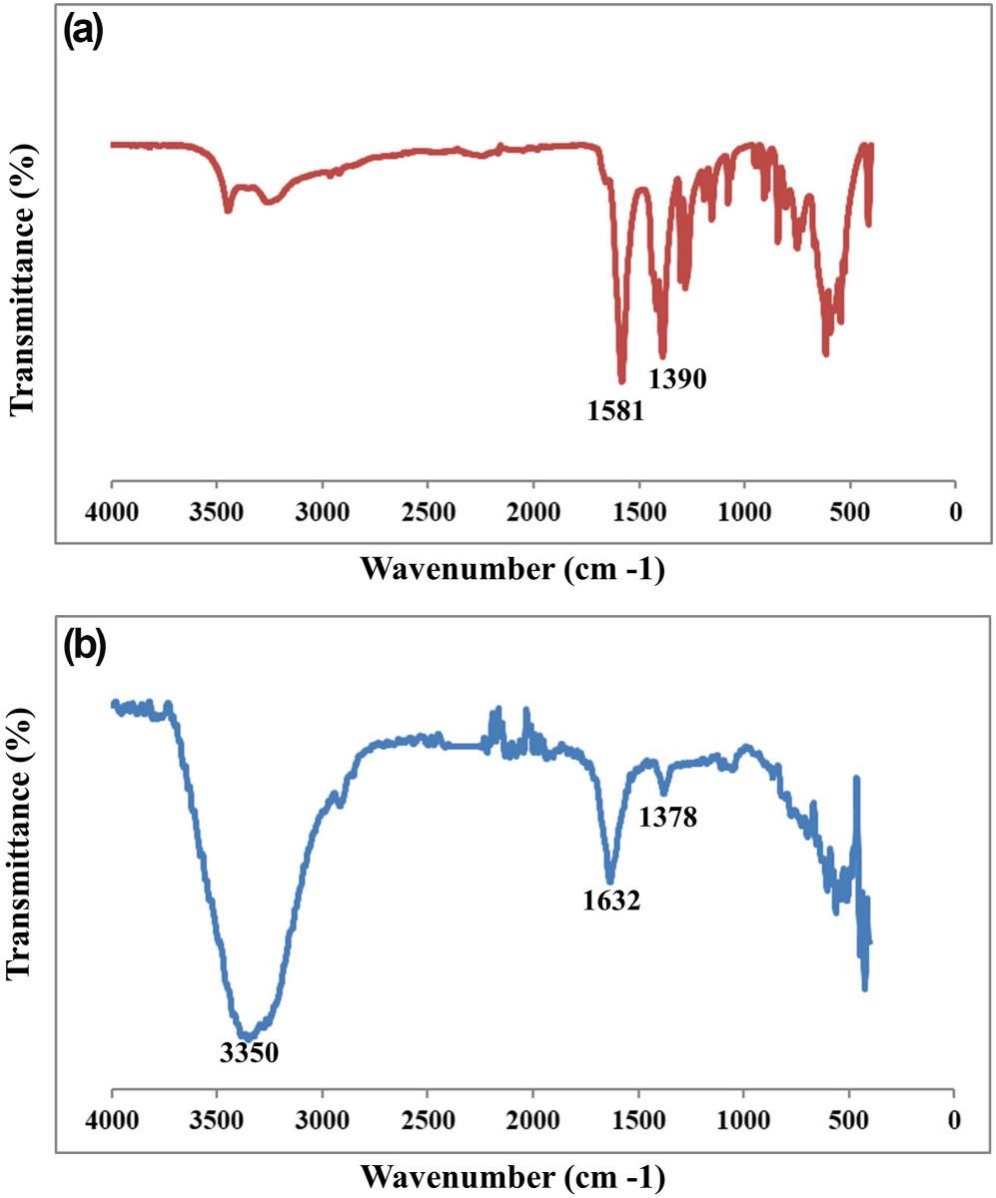
FTIR spectroscopy of citrate‐capped gold nanoparticles (AuNPs) synthesised in different reaction conditions, (a) Trisodium citrate powder and (b) citrate‐capped AuNPs.

FTIR spectroscopy was also applied to identify the characteristic functional groups of final citrated capped AuNPs obtained from optimisation synthesis experiments. All successful experiments provided similar characteristic peaks related to the citrate capping of AuNPs, as shown in Figure 4, despite FTIR curves of Na_3_Cit powder and synthesised citrated capped AuNPs samples. As put forward by previous studies, the presence of citrate capping is confirmed with remarkable peaks of 1395 cm^−1^ and 1586 cm^−1^, ascribed to the symmetric and anti‐symmetric stretching of the carboxyl group citrate structure respectively [26]. As shown in Figure 4a, these characteristic peaks were found at 1390 cm^−1^ and 1581 cm^−1^ in the FTIR spectrum of the Na_3_Cit powder sample, which was also obtained in FTIR curves of colloidal citrated capped AuNPs solution at peaks 1378 cm^−1^ and 1632 cm^−1^ (Figure 4b). In line with former findings, the FTIR spectra provided the hydroxyl groups stretching and bending bands on the surface of NPs at about 3350 cm^−1^ and 1632 cm^−1^ [30]. In detail, the clear peak at 1632 cm^−1^ of colloidal citrated capped AuNPs solution, as reported at 1639 cm^−1^ in similar studies [22], indicates the overlapping of peaks between anti‐symmetric stretching of the carboxyl group of citrate structure at the range of 1580–1590 cm^−1^ and the bending vibrational bands of hydroxyl groups, at the range of 1620–1650 cm^−1^ corresponding to water molecules inside the solution or on NPs' surface [22, 31].

### 3.2 Effect of initial reaction temperature

The relationship between a range of initial reaction temperature, 25, 55, 65, 75, 85, and 95°C, final particle size determined by DLS and maximum SPR absorption, and the laboratory images of the synthesised samples at different temperatures are presented in Figure 3. The results indicate that this synthesis reaction requires heat so that synthesis does not occur at 25°C. The synthesised NPs were 12–19 nm and 13–29 nm in methods I and II respectively. It implies that the method I provided narrower size distribution and smaller size of AuNPs (Figure 3a). In addition, the reaction's initial temperature was inversely related to the size of NPs, which is in line with previous studies [18, 22]. The nucleation rate in the formation process of NPs depends on the presence of DCA in the reaction. The higher temperature increases the thermal oxidation rate of the Na_3_Cit, increasing the rate of DCA production and, subsequently, a more considerable amount of nucleation rate. Due to the same initial concentration of HAuCl_4_, more seed particles and smaller sizes are formed [18, 19, 22].

Moreover, the smallest size and maximum SPR absorption were observed at 95°C in both methods I and II. The lowest absorbance and the most significant size were obtained at 55°C, consistent with previous results [22, 32]. Although rising temperatures directly affect NP size, the initial temperature hardly affected maximum SPR absorption, implying the synthesis efficiency. Temperature variations have little effect on SPR, which is in line with previous studies [22, 32].

### 3.3 Effect of Na_3_Cit/HAuCl_4_ molar ratios

**FIGURE 5:**
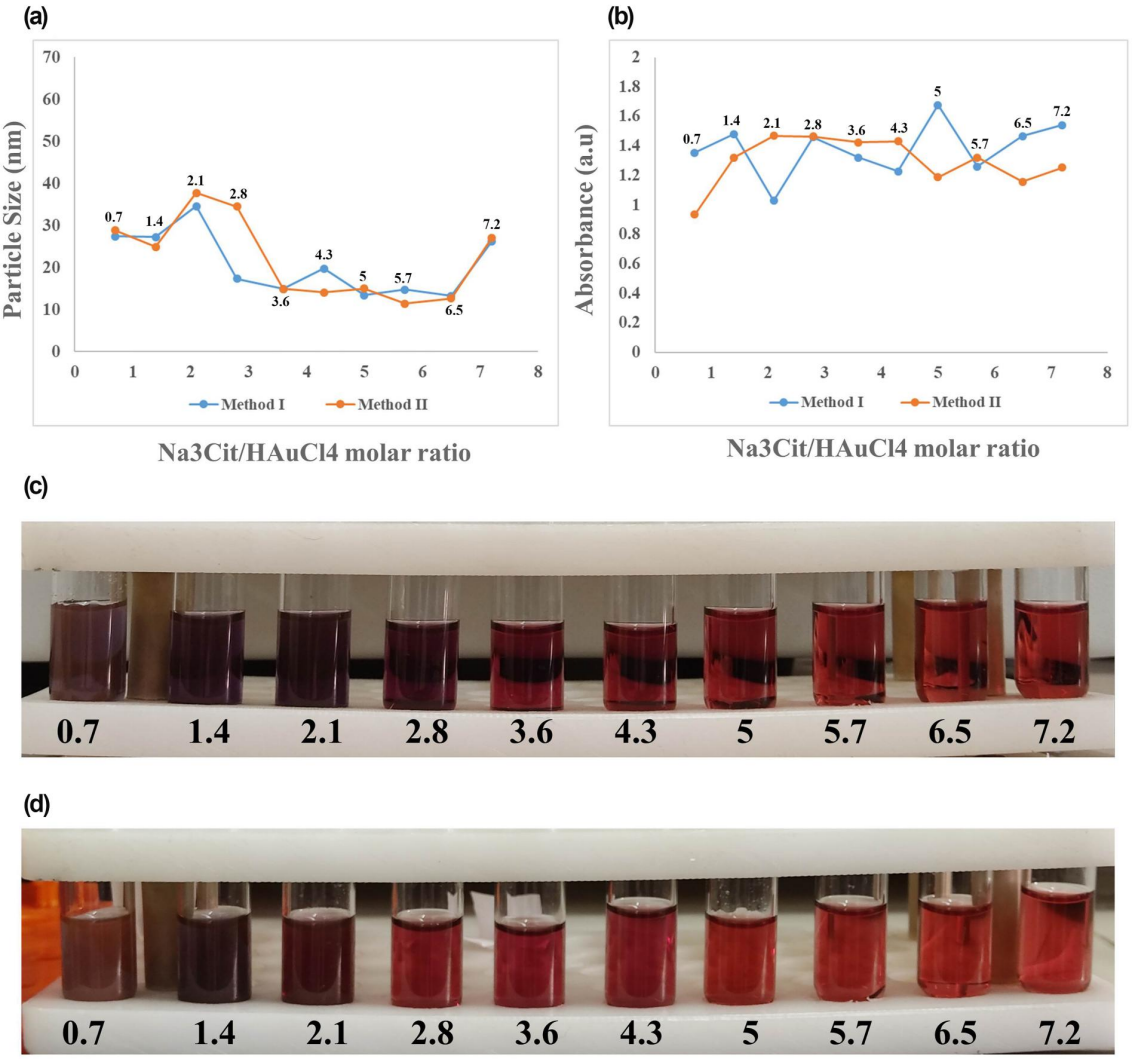
Effect of Na_3_Cit/HAuCl_4_ molar ratios 0.7–7.2 on (a) final particle size and (b) maximum SPR absorption of gold nanoparticles (AuNPs) synthesised by two methods, along with the laboratory images of NPs obtained by (c) method I and (d) method II. SPR, surface plasmon resonance

The effect of different molar ratios of Na_3_Cit/HAuCl_4_ on the size distribution determined by DLS and SPR absorbance of final synthesised NPs is shown in Figure 5, in both methods I and II with molar ratios of 0.7–7.2 along with the relevant laboratory images of the prepared samples. The size distribution of prepared NPs was achieved by 13–35 nm in method I and 11–38 nm in method II. The results did not indicate a significant difference between the two methods in size distribution among the examined Na_3_Cit/HAuCl_4_ molar ratios (Figure 5a), though the smaller size was obtained by method II. The higher Na_3_Cit/HAuCl_4_ molar ratio and thus higher concentration of Na_3_Cit led to the smaller size of final NPs, which is in line with the results of previous articles [18]. Furthermore, the smallest sizes of synthesised NPs were 11–13 nm, related to the Na_3_Cit/HAuCl_4_ molar ratio of 5–6.5 in both methods (Figure 5b). In the method I, the size of 13 nm was obtained in the Na_3_Cit/HAuCl_4_ molar ratio of 5 and 6.5, and the optimum molar ratio seems to be 5 due to the higher maximum in the SPR absorbance (1.67). Additionally, method II presented the smallest size of NPs in the Na_3_Cit/HAuCl_4_ molar ratio of 5.7, which was considered the optimum molar ratio for this method with an acceptable maximum SPR absorbance (1.3).

The higher molar ratio of Na_3_Cit/HAuCl_4_ leads to more presence of DCA in the reaction medium, responsible for more nucleation and resulting from smaller size formed AuNPs. Since DCA has both reducing and stabilising properties, the seed growth phase stops sooner, and a smaller particle size will be achieved [19]. However, in the Na_3_Cit/HAuCl_4_ molar ratio of less than 3, the seeds aggregate due to the insufficient amount of reducing and stabilising agent in the environment, and larger particle sizes were obtained [22].

As the smaller size of final citrated capped AuNPs was obtained in the presence of the higher concentration of Na_3_Cit, our findings presented the limited effects of Na_3_Cit concentration on the reduction of final NPs' size. As shown in Figure 5a, the Na_3_Cit/HAuCl_4_ molar ratio of more than 6.5 could provide a larger size of final NPs than less concentration, more than the mean size of 20 nm. However, Sivaraman et al. presented the size reduction of AuNPs to less than 10 nm by increasing the concentration of Na_3_Cit in Na_3_Cit/HAuCl_4_ molar ratio up to 20 [33]. The rising Na_3_Cit concentration towards a smaller size is limited to some factors that need future studies.

### 3.4 Effect of the initial pH value of the reaction

**FIGURE 6:**
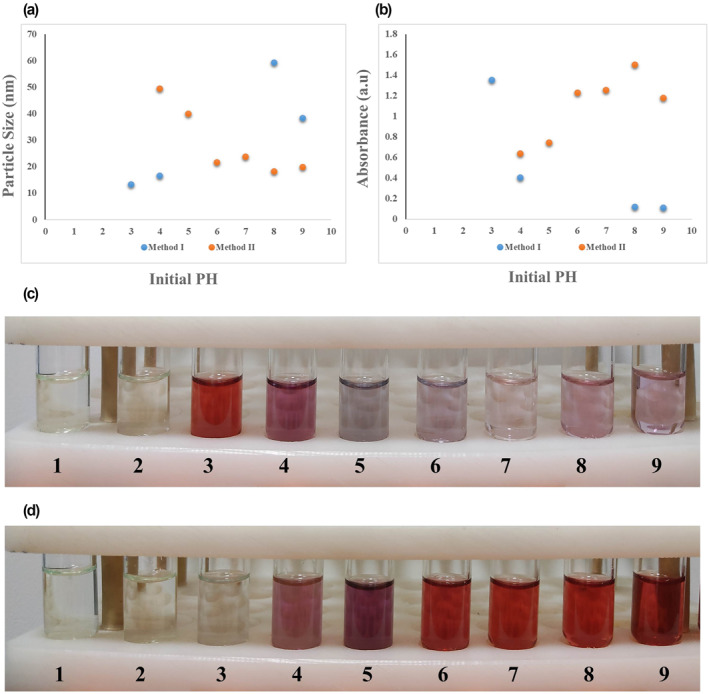
Effect of initial reaction pH values of 1–9 on (a) final particle size and (b) maximum SPR absorption of gold nanoparticles (AuNPs) synthesised by two methods, along with the laboratory images of NPs obtained by (c) method I and (d) method II. SPR, surface plasmon resonance

The initial pH value of the reaction is considered a determinative factor in the AuNPs synthesis process, size distribution, and efficacy. Figure 6 depicts the effect of different initial pH ranges of 1–9 on the size distribution and maximum SPR absorption of final synthesised AUNPs, in both methods I and II. As illustrated in the results, AuNPs fail to be synthesised in initial pH < 3 by both methods of I and II, which indicates the significant role of pH management in this reaction. The size distribution of synthesised NPs was 13–60 nm in method I and 15–50 nm in method II. Moreover, the smallest size of AuNPs was obtained from pH: 3‐4 and pH: 7‐8 in methods I and II respectively. It indicates that method II provides narrower size distribution though the smaller size was obtained by method I (Figure 6a).

Among designed experiments led to successful synthesis by the method I (initial pH = 3, 4, 8, and 9), experiment with initial pH = 3 and 4 offered a mean particle size of under 20 nm, while it was above 20 nm in final NPs of experiments pH = 8 and 9. Given that the maximum SPR absorbance of >1 in pH = 3 compared to ≤0.4 in pH = 4, 8, and 9, the experiment designed with initial pH = 3 presents a more desirable size and efficacy in the method I AuNPs synthesis. Dong et al. also reported the higher initial pH value of AuNPs synthesis reaction resulting in decreasing the reactivity of HAuCl_4_ complex and minor AuNPs fabrication [22].

On the other hand, method II presented a successful synthesis of AuNPs in initial pH = 4–9. Moreover, in this method, AuNPs obtained from synthesis experiments of initial pH = 6–9 showed a relatively smaller size than pH = 4 and 5 with higher maximum SPR absorbance >1. The experiments with initial pH = 6–9 could present the more efficient synthesis and desirable mean size of AuNPs (mean particle size <25 nm) by method II. In detail, pH = 7‐8 showed a mean particle size <20 nm. Similar results have also reported the fabrication of AuNPs in reverse, adding reagents order of Turkevich method in previous studies. It found that improvements in the size distribution, uniformity, and reproducibility of final synthesised NPs could be achieved by optimising reaction conditions, controlling initial pH at a lower value of ∼5.5, and high Na_3_Cit/HAuCl_4_ ratio [34].

### 3.5 Effect of reagent addition order

As noted previously, the order of reagent addition plays the leading role in the synthesis process and final characteristics of NPs. In this context, some studies applying reverse order of reagent adding of Turkevich method claimed the smaller sizes and narrower size distribution of final NPs compared to typical approach, which could be related to more reactivity of HAuCl_4_ in acidic environments during its adding time to reaction [33]. Our findings hardly significantly differed in size distribution, and the SPR absorption of NPs obtained from the two methods. However, the final smaller sizes were related to method II, in different molar ratios of Na_3_Cit/HAuCl_4,_ and method I understudied synthesis conditions based on manipulating initial temperature and pH values (Table 1). As proposed in previous works, once Na_3_Cit injects into the HAuCl_4_ solution in the Turkevich method, the initial pH value is about 3–3.5, which suddenly rises after Na_3_Cit addition due to its buffer functions. While in the reverse approach of adding reagents order, the low pH of HAuCl_4_ (pH = 1.6) in its adding time to boiling Na_3_Cit solution makes it more reactive and consequently improves the nucleation phase.

**TABLE 1:** The impact of various ranges of experimental parameters including initial pH value, initial temperature and Na_3_Cit/HAuCl_4_ Molar ratio on size distribution and maximum SPR absorbance of synthesised citrated‐capped AuNPs

Synthesis parameters	Order of reagent addition	Initial pH value	Initial temperature (°c)	Na_3_Cit/HAuCl_4_ Molar ratio	Size distribution (nm)	Maximum SPR absorbance
Temperature variation	Method I	3	25 & 55–95	5	12.86–18.83	0.99–1.48
	Method II				13.66–28.63	0.97–1.35
Molar ratio variation of Na_3_Cit/HAuCL_4_	Method I	3	95	0.7–7	13.27–34.6	1.03–1.67
	Method II				11.46–37.73	0.93–1.46
pH value variation	Method I	1–9	95	5	13.43–59.3	0.11–1.35
	Method II				15–49.6	0.64–1.50

Abbreviation: SPR, surface plasmon resonance.

Additionally, the reverse approach gives Na_3_Cit enough time to convert into DCA from thermal oxidation during the boiling process. This approach also eliminates the induction phase before the reaction with added HAuCl_4_. In addition, more effective stabilisation occurs in the neutral reaction medium, the initial pH of the reverse Turkevich method [19, 33].

### 3.6 Other factors in Gold nanoparticles synthesis approach

Further works have looked into the optimisation of AuNPs synthesis by considering other factors, including synthesis batch size and the latent heat of reaction. As reported by Dong et al., the characterisation of final AuNPs is not affected by Scaling up the synthesis, that batch sizes of 50 ml batch and 1.5 L provided no significant difference in final particle size, size distribution, or the optical NPs properties [22]. Moreover, Ding et al. proposed that the latent heat of boiling gold salt solution is one of the determining factors in forming AuNPs in the Turkevich method. Reduction of final particle size was observed up to 3 nm by increasing the latent heat due to increasing nucleation and growth rate during NPs synthesis reaction [35].

Taken together, the results point towards the substantial role of synthesis condition factors in the physicochemical properties of AuNPs, which highly affect their biomedical benefits. The particle size and shape of NPs significantly contribute to the pharmacokinetic properties of these NPs and the potential implications of their in *vivo* applications [36, 37]. The blood circulation, biodistribution, and clearance rate of AuNPs are highly associated with particle size, shape, surface functionalisation, and biotoxicity [37, 38]. Moreover, as mentioned previously, the smaller sizes of AuNPs, about 10–30 nm, present more tumour delivery due to greater penetration into cells and better anticancer effects, as the smaller size showed more extended blood circulation, which also helps to improve drug delivery and antimicrobial activities [11, 39]. In addition, a fast, facile, and cost‐effective method is needed to synthesise NPs. They are targeted for large‐scale productions as promising tools for biomedical applications [40, 41], thereby optimising convenient and short‐time synthesis approaches for AuNPs in terms of final particle size. Synthesis efficacy could improve future synthesis costs for large‐scale production in relevant dimensions. However, further studies will need to be undertaken.

### 3.7 Characterisation of optimised Gold nanoparticles

Gold nanoparticles synthesised under the optimised reaction conditions were evaluated for more detailed characterisation using UV/Visible spectroscopy, TEM, and XRD analysis. The optimised synthesis conditions were suggested as a temperature of 95°C, Na_3_Cit/HAuCl_4_ Molar ratio of 5, and the initial pH values of 3 for method I and temperature of 95°C, Na_3_Cit/HAuCl_4_ Molar ratio 5.7, and the initial pH values of 7‐8 for method II.

**FIGURE 7:**
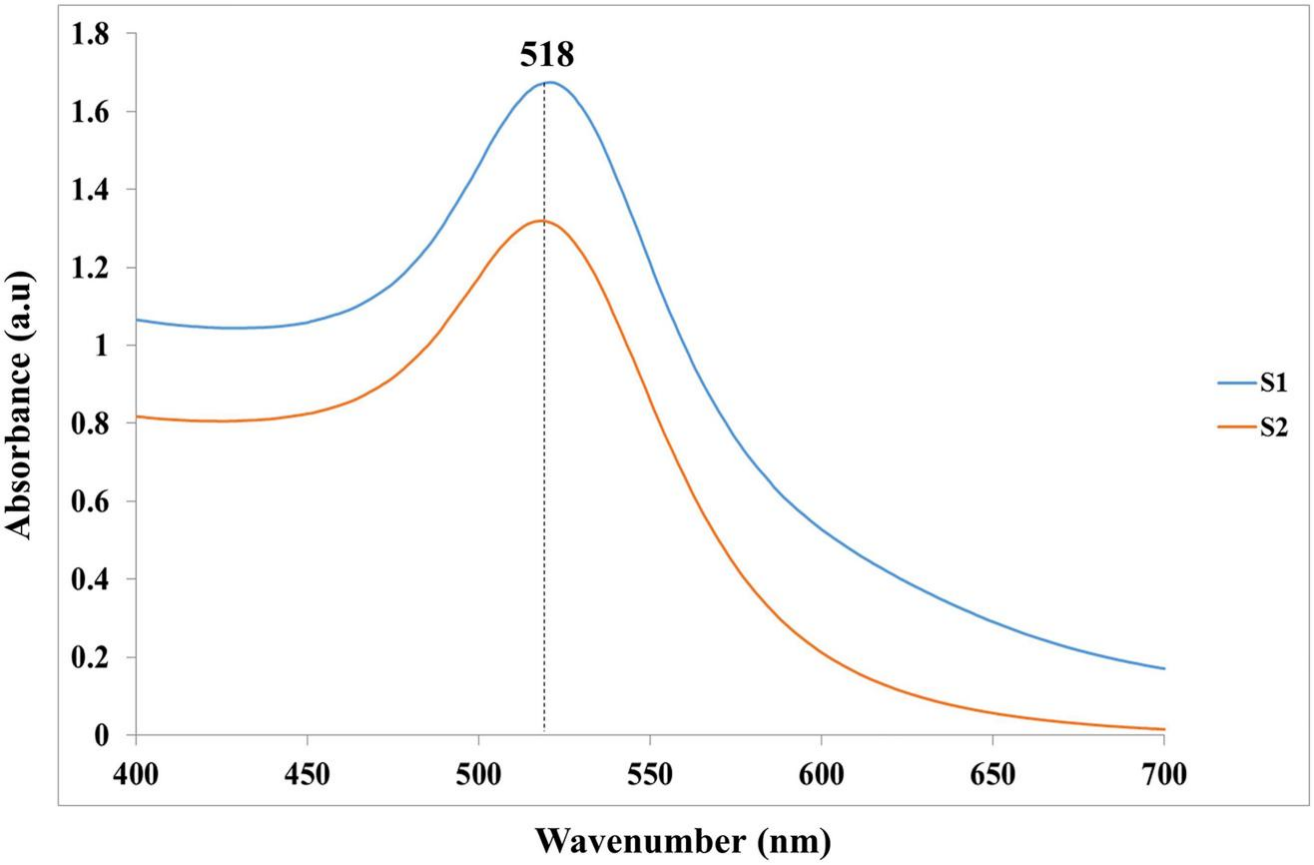
UV‐Vis absorption spectra of citrate‐capped gold nanoparticles (AuNPs) synthesised under optimised conditions of method I (S1) and method II (S2).

**FIGURE 8:**
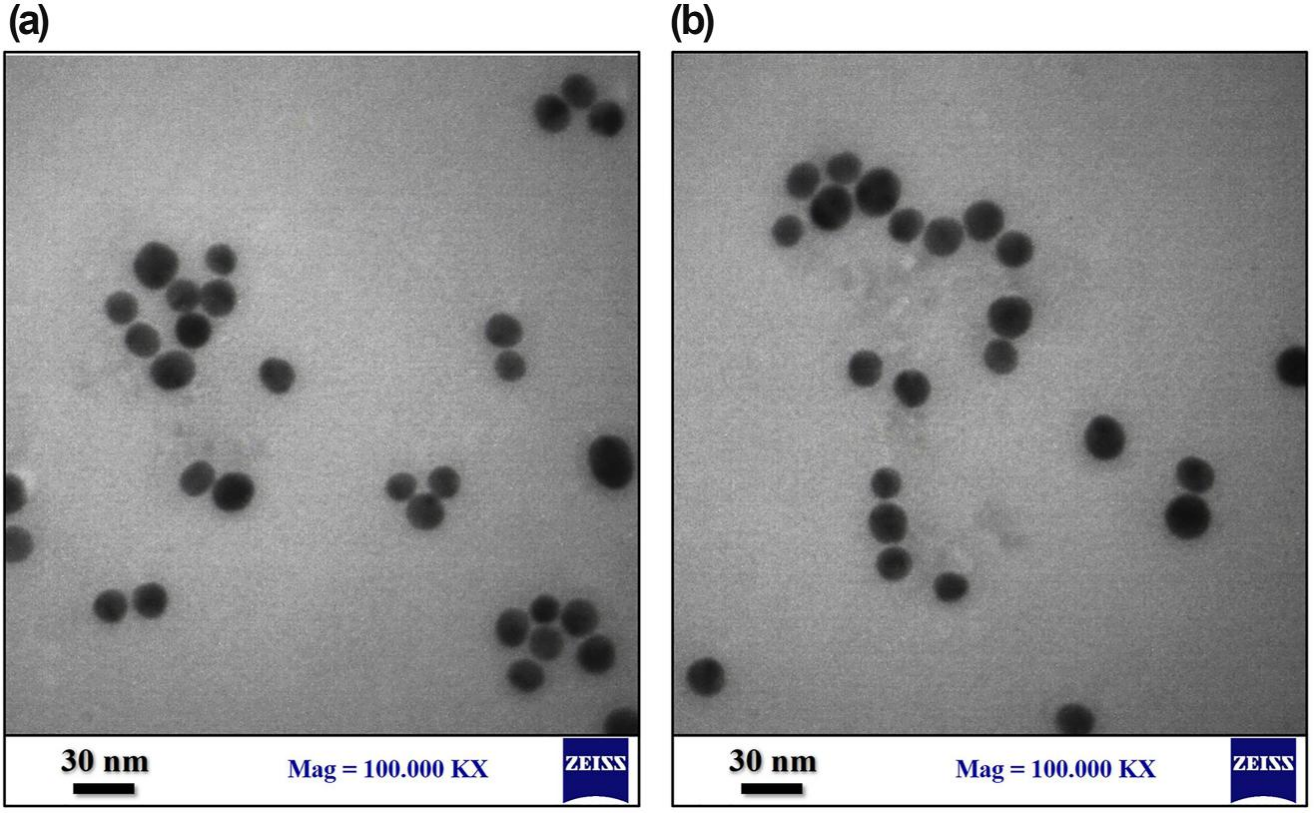
TEM micrographs of citrate‐capped gold nanoparticles (AuNPs) synthesised under optimised conditions of (a) method I (S1) and (b) method II (S2).

Figure 7 exhibited the UV absorption spectrums of citrated capped AuNPs synthesised under optimised conditions of method I (S1) and method II (S2). The results indicated that both NPs presented just a single SPR band at 518 nm, related to the characteristic SPR absorption of spherical AuNPs. Moreover, the TEM technique evaluated the morphology and particle size distribution of these NPs. As shown in Figure 8, the NPs possessed spherical morphology with good monodispersity and a final size of less than 15 nm, which is in line with the results of DLS data. These findings confirmed the effective impacts of the synthesis parameters considered optimised conditions on final NPs size and morphology.

**FIGURE 9:**
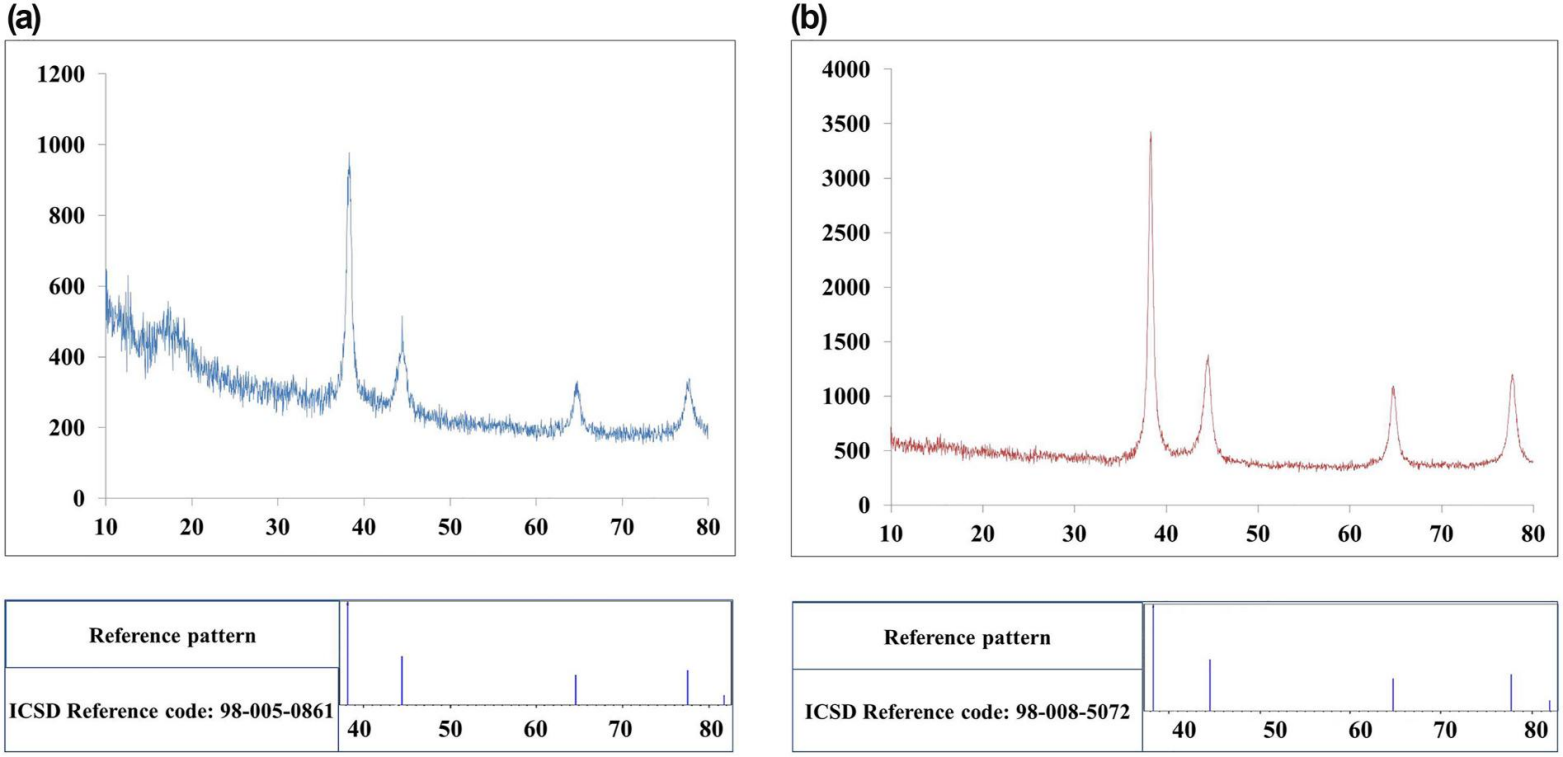
XRD patterns of citrate‐capped gold nanoparticles (AuNPs). synthesised under optimised conditions of (a) method I (S1) and (b) method II (S2).

In addition, XRD analysis was performed to evaluate the optimised NPs in terms of the crystallinity structure and crystallite size. According to the analysis of XRD data of optimised NPs (Figure 9), the Au atoms resulting from the reduction of Au^3+^ ions during the nucleation phase of the synthesis process remarkably formed the monodisperse crystals of the cubic phase in S1 and S2 nanostructures. The XRD patterns for S1 (Figure 9a) presented a few sharp peaks at 38.22°, 44.43°, 64.68° and 77.58°, which are ascribed to the characterisation peaks of cubic phase AuNPs, inorganic crystal structure database (ICSD) reference code: 98‐005‐0861. Besides, the results of S2 exhibited very similar characteristics to S1 with clear peaks at 38.26°, 44.51°, 64.71°, and 77.62° corresponding to the ICSD reference code: 98‐008‐5072 of cubic phase nano‐sized gold. Moreover, the crystallite size was close to that obtained from the DLS test and TEM, 14.5 and 18.3 nm of S1 and S2 respectively. These results could indicate the single crystal of each NP, one of the significant factors in synthesis optimisation studies that could be considered in further studies.

## 4 CONCLUSION

Economic approaches to AuNP synthesis are crucial to be applied in large‐scale productions. Accordingly, some studies have recently concentrated on optimising these potential NPs headed for various applications. The current study designed various experiments to optimise AuNPs synthesis with final desirable size distribution and efficacy by considering different reaction conditions. Our findings indicated that an increase in initial temperature of reaction and Na_3_Cit/HAuCl_4_ Molar ratio tend to obtain smaller particle size with more synthesis efficacy in both reagent addition orders, particularly in method I. Furthermore, the results highlighted the critical role of pH values and order reagent addition in final particle characteristics.

In summary, the optimum synthesis conditions for achieving NPs with smaller final particle size along with the increased synthesis efficacy considered temperature of 95°C, Na_3_Cit/HAuCl_4_ Molar ratio of 5 and 5.7, and the initial pH values of 3 and 7‐8 in the method I and II respectively. Besides, the optimised NPs showed highly crystalline structures, consisting of single crystals in the cubic phase along with crystallite size to particles. To sum up, it seems that the synthesis parameters play vital roles in reaction efficacy and final NPs characteristics, which is consistent with previous studies. As the necessity of synthesis optimisation in the direction of cost‐effective large production with an applicable size distribution, further works are needed to consider dedicated applications.

## AUTHOR CONTRIBUTIONS

**Zahra Bahmanyar**: Investigation; Project administration; Writing – original draft. **Fatemeh Mohammadi**: Investigation; Methodology; Visualisation; Writing – original draft; Writing – review & editing. **Ahmad Gholami**: Conceptualisation; Methodology; Project administration; Supervision; Writing – review & editing. **Mehdi Khoshneviszadeh**: Conceptualisation; Supervision; Writing – review & editing.

## CONFLICT OF INTEREST

The author declares that there is no conflict of interest that could be perceived as prejudicing the impartiality of the research reported.

## Data Availability

The data that support the findings of this study are available from the corresponding author upon reasonable request.
